# Contextualized Small Target Detection Network for Small Target Goat Face Detection

**DOI:** 10.3390/ani13142365

**Published:** 2023-07-20

**Authors:** Yaxin Wang, Ding Han, Liang Wang, Ying Guo, Hongwei Du

**Affiliations:** 1 College of Electronic Information Engineering, Inner Mongolia University, Hohhot 010020, China; 32156106@mail.imu.edu.cn (Y.W.); 32256150@mail.imu.edu.cn (H.D.); 2 State Key Laboratory of Reproductive Regulation and Breeding of Grassland Livestock, Hohhot 010020, China; 3 Department of Electronic Engineering, College of Information Science and Engineering, Fudan University, Shanghai 200438, China; 4 College of Information Engineering, Inner Mongolia University of Science and Technology, Baotou 014010, China; gy_imu@163.com

**Keywords:** goat face detection, small targets, intelligent management systems

## Abstract

**Simple Summary:**

Goat identification is highly demanded in modern livestock management, and sheep face detection is an important basis for goat identification, for which we developed a new computer model that overcomes the challenges of unclear images, small targets, and low resolution. By considering the surrounding details and combining different features, our model performs better than existing methods in detecting goat faces. We used various evaluation metrics to measure its effectiveness and found a significant improvement in accuracy. The results confirmed that our method successfully addresses the difficulty of detecting lamb faces. This study has important implications for the development of intelligent management systems for modern livestock farms to better identify and monitor goat for improved animal welfare.

**Abstract:**

With the advancement of deep learning technology, the importance of utilizing deep learning for livestock management is becoming increasingly evident. goat face detection provides a foundation for goat recognition and management. In this study, we proposed a novel neural network specifically designed for goat face object detection, addressing challenges such as low image resolution, small goat face targets, and indistinct features. By incorporating contextual information and feature-fusion complementation, our approach was compared with existing object detection networks using evaluation metrics such as F1-Score (F1), precision (P), recall (R), and average precision (AP). Our results show that there are 8.07%, 0.06, and 6.8% improvements in AP, P, and R, respectively. The findings confirm that the proposed object detection network effectively mitigates the impact of small targets in goat face detection, providing a solid basis for the development of intelligent management systems for modern livestock farms.

## 1. Introduction

The rapid development of deep learning has provided transformative abilities for computer vision, and its application provides new directions for accomplishing tasks such as image feature extraction and recognition. Changes in computer vision and artificial intelligence technologies have led to the application of target detection technologies in a wide range of industries. Computer vision has gained wide applications in the field of face recognition, and its applications in personal identification and information verification are very fast and efficient. Agriculture 4.0 is furthering the application of deep learning methods in all areas of animal husbandry [1], and the implementation of individual goat identification helps with individual behavior analyses [2,3], disease prevention [4], and the precise management of goats [5]. Since faces contain rich textural information as well as unique facial features, are unique and universal, and are relatively easy to capture, facial detection is of high research value as an application of deep learning in agriculture and provides the basis for subsequent recognition and classification tasks.

Guo [6] was able to achieve 91.1% accuracy by using Faster R-CNN to detect primate faces, and then using Tri-attention to recognize the detected images. Hitelman [7] used the Faster R-CNN algorithm to locate the face of a goat in an image, analyzed and compared this using several classification models and migration learning methods, and finally obtained an accuracy of 97% using the ResNet 50 V2 model with ArcFace loss function. Li [8] et al. demonstrated that Vision Transformer can be applied to sheep facial recognition and proposed a lightweight MobileViTFace model combined with MobileNetV2, which can reach 97.13% accuracy.

Due to the habits of goats and the limitations of pasture monitoring, the captured goat face targets are usually small and clustered, which makes goat face target detection very difficult. There are several methods for small-target detection, as follows:

Supplementary contextual information, which can compensate the problem of limited features being extracted from small targets by supplementing the network with more contextual information related to small targets [9]. Leng et al. [10] developed a new inner and outer recurrent neural network (IENET) and designed a bi-directional feature fusion module (BI-FFM), a contextual inference module (CRM), and a contextual feature enhancement module (CFAM). FASSD [11] obtained contextual information by adding a feature fusion module to SSD and combining this with the attention module to improve the detection of small targets.

With respect to super-resolution techniques, Li [12] first proposed a perceptual GAN model for small target detection, which employs a deep residual network as a generator that enables super-resolution display and captures more details.

Loss functions are beneficial for the class-balancing of small objects and Liu et al. [13] proposed a new feedback-driven loss function that trains the model in a more balanced manner by feeding back information about the loss distribution.

In our goat face small-target dataset, the following difficult points needed to be resolved.

(a) The image resolution is too low, lacking sufficient feature information for recognition, and environmental factors can easily affect the detection effect;
(b) The number of positive samples for small targets is small and, when the boundary between the Anchor set by the model and the small target is large, the number of positive training samples for small targets will be much smaller than the number of positive samples for large targets; the model is prone to ignore the detection of small targets, especially when the target object spans a large scale;
(c) When performing feature fusion, the small target information is easily lost due to the small target features, which are easily overwhelmed by background noise.

To solve the above difficulties, we proposed a Contextualized Small Target Detection Network (CSTDNet). The main aspects of our network are as follows:

(i) A Contextual Information Detection Module (CIDM) was proposed, which can provide more background information and further contextual information about the target, which can help in the detection of small targets;
(ii) A Feature Complementary Module (FCM) was proposed to fuse the information of each scale while eliminating noise and interference, thus improving the reliability and anti-interference abilities of the feature target;
(iii) WH-CIoU was proposed on the basis of CIoU, which can calculate the difference between the predicted frame width and height relative to their true values. The loss function is more biased to the change in the prediction frame size, which is more favorable to the regression of the prediction frame.

## 2. Materials and Methods

This section introduces the definition of small targets and the network model used to perform small-target detection.

### 2.1. Small Target Definition

The definitions of small targets are divided into two categories according to the scale and size of the target objects: relative definition and absolute definition. Relative definition means that the size of the target is smaller than a certain percentage of the original dataset image; for example, the small-target objects in the Stanford Drone dataset [14] are smaller than 0.2% of the original image size and the median value of the relative area (the ratio of the area of the bounding box to the area of the image) of the defined small target in the PASCAL-VOC dataset [15] is less than 5%. The absolute definition specifies that the pixels of the target must be less than a certain value to be defined as a small target, such as the AI-TOD [16] dataset, which defines 8–16 pixels as a tiny target and 16–32 pixels as a small target; the DIOR [17] dataset defines the width or height of a small target as less than 50 pixels.

Based on specific applications and research scenarios, different definitions of small targets have been given by researchers for specific datasets. Referring to the face detection dataset WIDER FACE [18], which defines the scale of small targets for faces as 10–50 pixels, and the daily items SDOD-MT [19] dataset, which similarly defines the range of small targets as 10–50 pixels depending on the length of the horizontal bounding box, we specified that targets must be smaller than 50 pixels in the goat face dataset to be classified as small-target goat faces.

### 2.2. Network Architecture Design

The development of deep learning has allowed for target detection networks to excel in the field of target recognition, and the YOLO algorithm is a typical one-stage target detection algorithm. In this study, we used YOLOV7 [20] as the backbone network, aiming to improve it and achieve the target detection of goat faces.

#### 2.2.1. YOLOV7-Based Network Architecture

The YOLOV7 network model is an improved target detection network based on the YOLOV5 [21] optimization, using a feature pyramid FPN + PAN structure to fuse features from different feature layers, which is beneficial for feature extraction. The network uses the SPPCSPC module; the SPP module obtains different perceptual fields by maximum pooling, which can increase the perceptual field and allow the algorithm to adapt to different resolution images. The CSP module first divides the features into two parts, one of which is processed conventionally while the other is processed by the SPP structure, and later merges the two parts, which can reduce the computation time by half. This enables the speed to become faster and the accuracy to be improved. In terms of sample assignment strategy, YOLOV7 combines the positive and negative sample assignment strategies in YOLOV5 and YOLOX [22], which can provide more accurate prior knowledge.

#### 2.2.2. Network Structure

**Figure 1:**
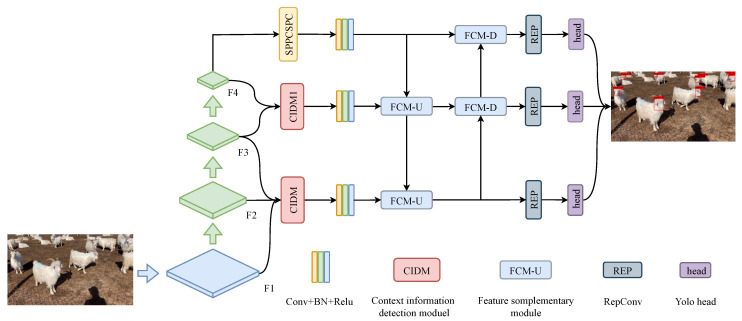
Contextualized Small-Target Detection Network (CSTDNet). The CIDM1 in the figure is a CIDM without the line1 in Figure 2.

The network structure (Figure 1) of this paper consists of backbone, neck and prediction. Backbone is the same as the backbone part of YOLOV7, and the neck part consists of a contextual information detection module, feature-fusion complementary module, and other modules, which will be described in detail below. The detection head in prediction is the same as that in YOLOV7, using three YOLO heads for detection. Since the detection type is only one category of goat face, the shape of the three feature layers in the detection head are (20, 20, 18), (40, 40, 18), and (80, 80, 18). The last shape of 18 can be split into three sixes, corresponding to the six parameters of the three prior boxes, while six can be split into four + one + one. Four is the regression parameter of each feature point, and adjusting the regression parameter can obtain the prediction framework; the first one determines whether each feature point contains an object, and the second one determines the feature category, because there is only one category, so it is one.

### 2.3. Context Information Detection Module

The detection of small goat face targets presents a formidable challenge due to multiple factors. Notably, acquired goat face targets exhibit blurriness, inconspicuous features, and considerable environmental interference. Furthermore, these small goat face targets typically occupy only a few pixels within the overall image, further exacerbating their susceptibility to noise, texture, and other disruptive factors. Consequently, accurately identifying and locating these small targets becomes arduous. To address the issue of substantial interference and the resulting low detection accuracy in goat face small target detection, we proposed a contextual information detection module. This module leverages the contextual information surrounding the target, enabling the acquisition of additional background information and contextual semantic cues. By incorporating such contextual information, the proposed module offers tangible benefits for the detection and localization of small targets.

**Figure 2:**
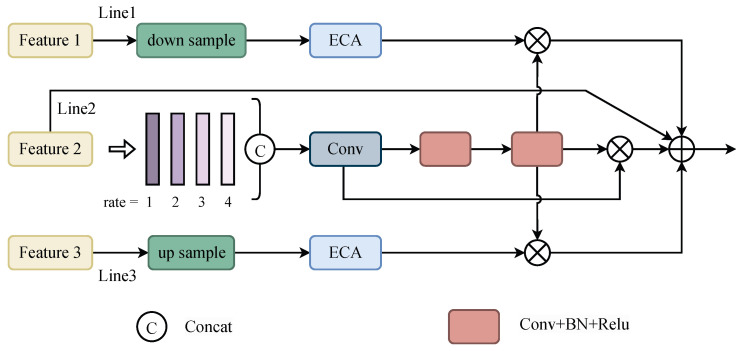
Context information detection module.

We used a dilated convolution with different convolution rate sizes to form different receptive fields and extract local contextual information. As shown in the local context extraction backbone in Figure 2, a multi-branch convolutional block exists, in which each branch will extract information from different perceptual fields. We used dilated convolution with convolutional rates of 1, 2, 3, and 4 to extract information; to extract more information, the convolutional kernel size was increased to 3×3, and the extracted information was fused by a cat operation to form local contextual information.

(1) $$F_{2}=CAT\left(Conv_{\left(k=3,p=i,r=i\right)}\left(F_{2}\right)\right),i=1,2,3,4,$$

where CAT(·) is concatenation and $Conv_{\left(k=3,p=i,r=i\right)}\left(\cdot \right)$ is the dilated convolution with different convolution rates.

Local contextual information often exists in a feature layer. In order to obtain global contextual information in the surrounding environment, it is necessary to combine feature information from adjacent feature layers; therefore, we introduced feature interactions between features at adjacent levels and, since the previous and subsequent features have different scales to the current feature, the two adjacent branches were, respectively, upsampled and downsampled after convolution to align the number of channels. Then, the extraneous information generated in the feature interactions was suppressed by the ECA attention module [23] to reduce the sensitivity to noise and interference.

(2) $$F_{1}=Conv\left(ECA\left(Up\left(F_{1}\right)\right)\right),$$

(3) $$F_{3}=Conv\left(ECA\left(Down\left(F_{3}\right)\right)\right),$$

where Up(·) and Down(·) are upsampling and downsampling operations, respectively, and ECA(·) is the ECA attention mechanism, an efficient attention module.

The final output is *F*,

(4) $$F=F_{1}+F_{2}+F_{3}.$$

### 2.4. Feature Complementary Module

**Figure 3:**
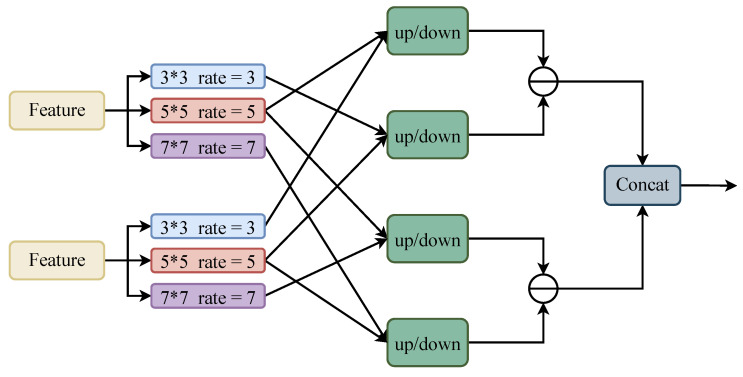
Feature complementary module.

To fuse the feature maps generated at the different stages, we proposed FCM-U and FCM-D, as shown in Figure 3. FCM-U aggregates the three features generated by the contextual information detection module and SPPCSPC and aligns the number of channels to improve the feature resolution and generate a new feature map in FCM-D to fuse the features generated in the previous stage. In FCM-U and FCM-D, we used convolution kernels of 3, 5, and 7 for dilation convolution to receive the correct number of channels for feature alignment, and then divided these two by two into UP or DOWN modules to improve and reduce the feature resolution. Finally, a differencing operation was performed to fuse the features and suppress the background noise generated during feature-fusion by differencing.

**Figure 4:**
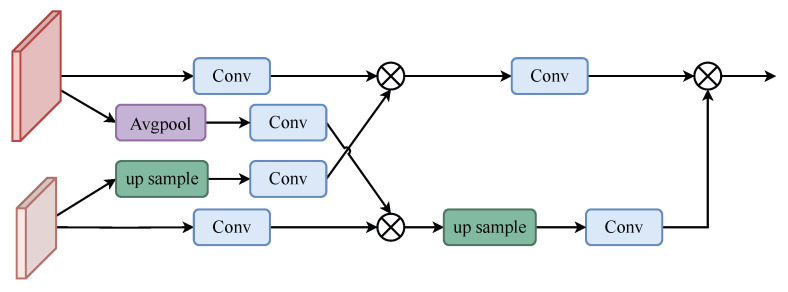
FCM-Up.

As shown in Figure 4, the high-resolution feature $f_{1}$ is downsampled by averaging pooling to obtain $down(f_{1})$, and the low-resolution feature $f_{2}$ is upsampled by bilinear interpolation to obtain $up(f_{2})$. $up(f_{2})$ and $down(f_{1})$ are then convolved 3×3 to obtain $Conv(down\left(f_{1}\right))$ and $Conv(up\left(f_{2}\right))$, respectively. The convolved $Conv(f_{2})$ is multiplied by $Conv(down\left(f_{1}\right))$ to obtain the feature $f_{12}$.

(5) $$f_{12}=Conv\left(down\left(f_{1}\right)\right)\times Conv\left(f_{2}\right).$$

Similarly, multiply the convolved $Conv(f_{1})$ with $Conv(up\left(f_{2}\right))$ to obtain the feature $f_{21}$.

(6) $$f_{21}=Conv\left(up\left(f_{2}\right)\right)\times Conv\left(f_{1}\right).$$

Finally, the upsampled $f_{12}$ is $up(f_{12})$ passed through the convolution block and multiplied with the convolved $f_{21}$, $Conv(f_{21})$ to obtain the output feature. The operation can be expressed as follows:

(7) $$f_{final1}=Conv\left(f_{21}\right)\times Conv\left(up\left(f_{12}\right)\right).$$

**Figure 5:**
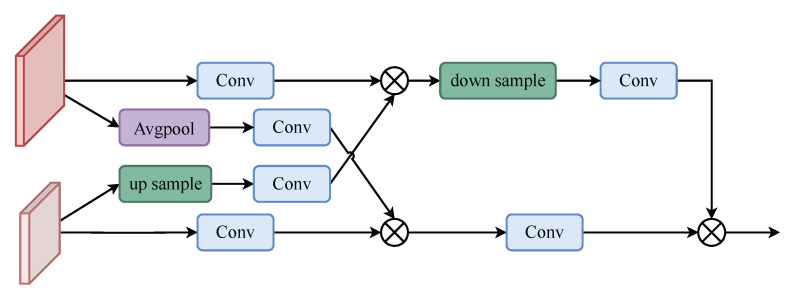
FCM-Down.

The down module is similar to the up module;as shown in Figure 5 the only difference is that, instead of upsampling $f_{12}$, $f_{21}$ is downsampled $down(f_{21})$ through the convolution block and multiplied through the convolution block with $f_{12}$ to obtain the output features. The operation is given in the following equation:

(8) $$f_{final2}=Conv\left(f_{12}\right)\times Conv\left(down\left(f_{21}\right)\right).$$

After up or down, the output features are set after the differential module to output the final feature results. The differential module can effectively offset noise and interference, thus improving the reliability and anti-interference ability of the feature target. The equation is as follows:

(9) $$difference=|F_{A}-F_{B}|.$$

$F_{A}$, $F_{B}$ are the output of the previous stage and ∣·∣ is the absolute value operation.

(10) OUT=CAT(difference1,difference2);

difference1 and difference2 are the outputs of difference in Figure 3, respectively, while CAT(·) is a concatenation operation.

### 2.5. Small-Target Detection Head and Loss Function

The anchor frame sizes of YOLOV7 were set to 12, 16, 19, 36, 40, 28, 36, 75, 76, 55, 72, 146, 142, 110, 192, 243, 459, and 401, which are very scientific for target detection but not suitable for our small-target goat face detection. In order to set the anchor frame at an appropriate size, we first performed a cluster analysis on the training set anchor frame size using the clustering algorithm. The maximum target to be detected was revealed to be 55 and the minimum was 8. Most of the edge labels of the target to be detected were concentrated from 24 to 32; therefore, we set the anchor frame sizes as 5, 9, 12, 16, 19, 36, 42, 31, 40, 28, 55, 48, 36, 75, 76, 55, 72, and 146 to improve the detection accuracy for small targets.

The values of the loss function of YOLOV7 include target confidence loss, category confidence loss, and coordinate regression loss.

(11) $$Loss=0.1\times L_{Con}+0.125L_{Cla}+0.05\times L_{Loc}.$$

Binary cross entropy is a common loss function used here to calculate confidence loss and category loss.

(12) $$L_{Con}=L_{Cla}=BCELoss=-\frac{1}{N}\sum \omega \times (gt\times ln(pr)+(1-gt)\times ln(1-pr)).$$

The localization loss $L_{Loc}$ can be calculated using IoU [24], GIoU [25], DIoU [26], and CIoU  [27] for loss calculation. IoU is the most commonly used metric in target detection, and can be used to evaluate the distance between the prediction frame and the ground true. The IoU formula is as follows:

(13) $$IoU=\frac{|gt\cap pr|}{|gt\cup pr|}.$$

GIoU differs from IoU in that it can focus on non-overlapping regions, and the GIoU formula is as follows:

(14) $$GIoU=IoU-\frac{|A_{c}-gt\cup pr|}{|A_{c}|}.$$

DIoU can focus on the distance, overlap and scale of the target and anchor; the equation of DIoU is as follows:

(15) $$DIoU=IoU-\frac{\rho^{2}\left(p_{pr},p_{gt}\right)}{d^{2}}.$$

CIoU is the addition of the detection box scale loss to DIOU; CIoU is as follows:

(16) $$CIoU=IoU-\frac{\rho^{2}\left(p_{pr},p_{gt}\right)}{d^{2}}-\alpha v,$$

where gt represents the ground-truth value, pr represents the prediction frame, and $A_{c}$ represents the area of the smallest closure region that contains both the prediction frame and the ground truth frame, $p_{pr}$ represents the centroid of the predicted frame, $p_{gt}$ represents the centroid of the ground truth frame, ρ is the Euclidean distance between the centroids of the truth frame and the predicted frame, d represents the minimum value of the diagonal of the region containing both the predicted frame and the ground truth frame, α is the weight function, and *v* is the parameter used to measure the consistency of the aspect ratio, which is given by the following equation:

(17) $$\alpha =\frac{v}{(1-IoU)+v}$$

(18) $$v=\frac{4}{\pi^{2}}(arctan\left(\frac{w_{gt}}{h_{gt}}\right)-arctan\left(\frac{w_{pr}}{h_{pr}}\right){)}^{2},$$

where $\frac{w_{gt}}{h_{gt}}$ represents the aspect ratio of the ground truth frame and $\frac{w_{pr}}{h_{pr}}$ represents the aspect ratio of the predicted frame.

CIoU takes into account the difference in the bounding box width-to-height ratio instead of the difference between the predicted box and the ground truth box width and height truth, which can hinder the model regression in some cases. When $\frac{w_{gt}}{h_{gt}}$ =$\frac{w_{pr}}{h_{pr}}$: when the predicted box width–height ratio is equal to the ground true box width–height ratio, the value of the αv term of CIoU is 0, which means that its penalty term will be useless and will degrade to DIoU. Based on the above, our CIoU was based on the proposed WH−CIoU, the width–height ratio was split and the variance in width and height relative to their true values were calculated separately, which can directly obtain the minimum value of the difference, which is more conducive to model convergence, and the WH−CIoU formula is as follows:

(19) $$WH-CIoU=IoU-\frac{\rho^{2}\left(p_{pr},p_{gt}\right)}{d^{2}}-\beta \epsilon$$

(20) $$\beta =\frac{\epsilon }{(1-IoU)+\epsilon }.$$

ϵ is as follows:

(21) $$\epsilon =\frac{2}{\pi^{2}}\left((arctan\frac{w_{pr}}{w_{gt}}-\frac{\pi }{4}{)}^{2}-(arctan\frac{h_{pr}}{h_{gt}}-\frac{\pi }{4}{)}^{2}\right).$$

$\frac{w_{pr}}{w_{gt}}$ is the ratio of the width of the prediction box to the width of the ground true box, $\frac{h_{pr}}{h_{gt}}$ is the ratio of the height of the prediction box to the height of the ground true box.

(22) $$L_{Loc}=1-IoU/GIoU/DIoU/CIoU/WH-IoU.$$

The individual IoUs were compared in the Section 3.

## 3. Experiments

This section describes the dataset used for the experiment, the evaluation metrics, and the results of the experiment.

### 3.1. Goat Face Image Dataset

In this paper, Albasian velvet goats were used as the test subjects and the data were collected in a pasture in Ordos, in the Inner Mongolia Autonomous Region in 2022. A 12-megapixel cell phone was used to shoot the video, with a frame size of 1920 pixels × 1080 pixels and a rate of 30 frames per second.

The test scenes included various scenes, such as goat barns and grasslands, and data were collected from hundreds of Albasian velvet goats. The data collection time range covered multiple time periods from 6:00 to 18:00 with different lighting conditions. The collected data covered different goat face images with different angles, and different poses were collected. The captured video was divided into independent goat images using the frame-splitting operation and one image was taken every 30 frames during the frame-splitting operation in order to prevent the appearance of overly similar images in the database.

**Figure 6:**
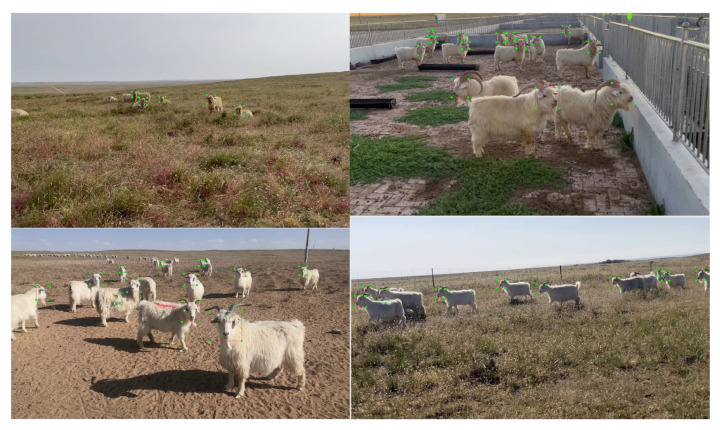
Small target goat face dataset.

After that, the images were filtered to keep high-quality images, remove blurred images, and remove images with high similarity. Filtered images were labeled with the goat faces using the Pascal VOC 2007 data format to complete the Albasian goat dataset, and then filtered by the clustering algorithm to remove the images containing goat faces larger than 50 pixel values. The images containing goat faces larger or smaller than 50 pixel values were then filtered by the clustering algorithm—the obtained target contained targets with 50 pixel values and below—and the small-target dataset of goat faces was obtained for our training.As in Figure 6 The dataset contained 8871 images with 65,894 goat faces.

**Figure 7:**
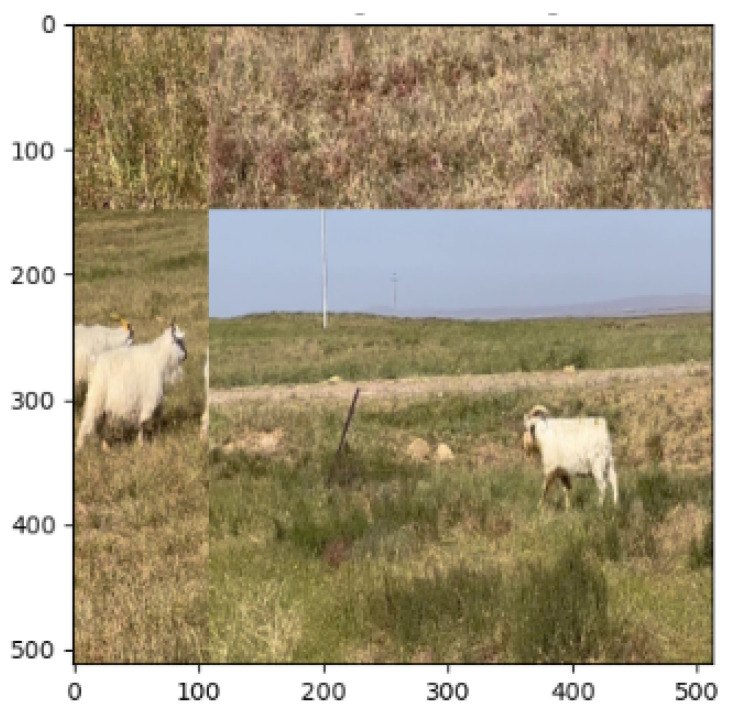
Mosaic data augmentation and Mixup data augmentation.

We used the Mosaic data augmentation method [28] and Mixup data augmentation method [29] to process the dataset.As in Figure 7, the mosaic data augmentation method can generate new training data by randomly combining multiple images. This method randomly scales, crops, and randomly distributes four images and then randomly stitches them into one large mosaic graph. This processing method can better enrich the dataset, while random scaling adds more small targets and makes the network more robust. The Mixup data enhancement method can generate new training data by randomly and linearly combining two different images. Specifically, it linearly mixes two images in a certain ratio to obtain a new image and linearly mixes their labels in the same ratio to obtain a new pair of labels.

### 3.2. Experimental Evaluation Metrics

To evaluate the performance of the proposed model more fairly and accurately, this paper used precision (*P*), recall (*R*), F1-Score(F1), and AP for the performance evaluation and comparison [30]. The formulae for each metric used in this paper are presented below:

1. Precision (P): This indicator describes the proportion of positive samples detected by the model that are actually positive. The larger the value, the higher the accuracy rate, which would ideally be 1:1:
  (23) $$P=\frac{TP}{TP+FP}.$$
2. Recall (R): This indicator describes the proportion of correct positive samples detected by the model among all positive samples:
  (24) $$R=\frac{TP}{TP+FN}.$$
3. F1-Score: This indicator combines precision and recall and takes a balanced value for a comprehensive assessment of:
  (25) $$F1=\frac{2\times P\times R}{P+R}.$$
4. AP: AP is the average precision, which is the area under the precision–recall curve:
  (26) $$AP=\int_{0}^{1}P\left(R\right)dR,$$
  where TP is the number of samples that are actually positive and predicted to be positive, FP is the number of samples that are actually negative but predicted to be positive, and FN is the number of samples that are actually positive but predicted to be negative.

Our goat face detection used bounding boxes to represent the position and size of the goat face. IoU is a method for measuring the position of bounding boxes, used to evaluate the degree of overlap between predicted and real bounding boxes, ranging from 0 to 1, where 1 represents complete overlap. Setting a certain threshold and retaining the bounding box can achieve the function of the detection of goat faces. The threshold used in this study was 0.7.

### 3.3. Test Environment and Network Parameter Settings

The designed model was trained using the Windows 10 64-bit operating system; the framework used for deep learning was PyTorch, the programming language was Python, and the computer had 16 GB of RAM with an AMD Ryzen 7 5800H with a Radeon Graphics processor and an NVIDIA GeForce RTX 3060 graphics card to accelerate image processing. The model was trained using batch training, with hyperparameters set to eight image samples per batch and an initial learning rate of 1 × 10${}^{-4}$. Using the ADAMW optimizer, the network model was operated to save weights every 10 completed iterations for a total of 100 iterations.

### 3.4. Comparison Experiments

To verify the accuracy of the models by comparison, we collected eight models that use achieved advanced methods in the field of target detection for comparison, including CenterNet [31], EfficientDet [32], SSD [33], FASSD [11], FCOS [34], RetinaNet [35], YOLOV5 [21], and V7 [20] of the same YOLO series. To ensure fairness in the experiments, we used source code as well as source evaluation methods for the experiments. The following Table 1 shows the experimental results of our proposed model and the comparison model for goat face detection.

Table 1 shows the best results obtained for goat face detection compared to the other detection models; AP improved by 8.07%, F1 improved by 0.06, and recall improved by 6.8%. In the statistical test section, we evaluated different algorithmic models using the Friedman test and the Nemenyi test to compare the performance of different methods on P, R, F, and AP metrics. We chose five models with excellent test performance to compare with our model. First, we made an assumption of model equality, named H. Table 1 shows the ranking results of these six models for different metrics. We calculated the chi-square value as $\chi^{2}$ = 14.429 and the *p*-value as *p* = 0.013 < 0.05. This indicates that the models show statistically significant differences between them. By performing the Nemenyi test, we can calculate the critical distance CD = 3.770. From this result, we can infer that our model CSTDNet is significantly different and performs better than SSD, YOLOV5. The effectiveness of CSTDNet for small target goat face detection was verified by comparing several methods through statistical tests. Table 2 shows the ranking of small target detection results based on comparative test data.

In the face of practical use, the image, in addition to the small target goat face, will also appear as a normal-sized goat face. In order to ensure the completeness of the experiment, while verifying the generalization ability of the model proposed in this paper, in the small target goat face test set after the normal-sized goat face test set was constructed once again, the size of the small target goat face data test set was the same as that of the normal-sized goat face test set, at 1006 sheets. Tests were performed on the constituent datasets, and the test results are shown in Table 3 below:

From Table 3, we can see that YOLOV7 is better than our model at normal size target detection, but our model still outperforms most of the models, which shows that ours possesses good generalization, outperforms YOLOV7 in small target detection, and achieves an approximately similar performance in normal size detection.

Similar to small target detection, the same Friedman test and Nemenyi test were used to evaluate six models, including ours, for normal-sized target detection. Table 4 shows the results of ranking these six models on different metrics for normal target detection. We calculated a chi-square value of $\chi^{2}$ = 18.813 and a *p*-value of *p* = 0.002 < 0.05. This indicates that there are statistically significant differences between the models. In the Nemenyi test, we calculated the critical distance, CD = 3.770, by which it can be inferred that our model CSTDNet is not significantly different from YOLOV7. A comparison of several methods using statistical tests verified that CSTDNet is equally effective in the detection of normal-sized goat face targets. Table 4 shows the ranking of normal-sized target detection results based on comparative test data.

**Figure 8:**
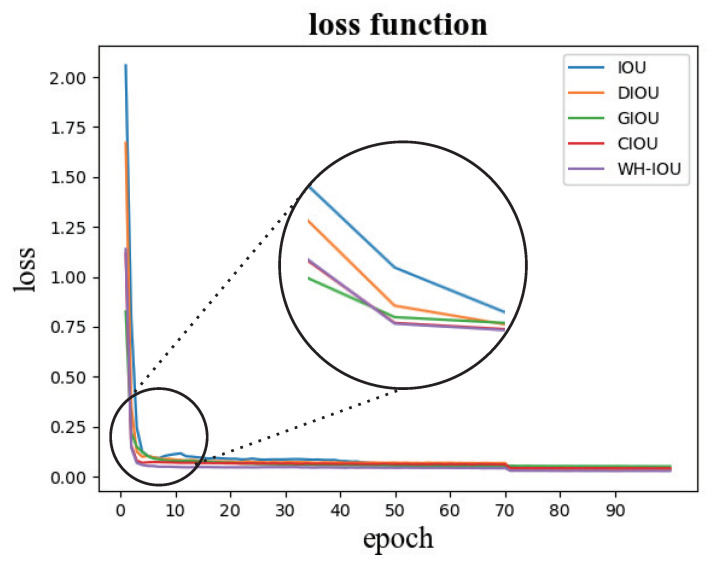
Loss function.

In Figure 8, the loss graphs obtained for each network on the goat face dataset are presented. As shown in Figure 8, our modified loss converged faster in the overall detection process, in addition to having a greater compensation mechanism for the detection of small targets.

### 3.5. Ablation Experiments

To demonstrate the effectiveness of the proposed modules of this model, ablation experiments were conducted and the evaluation dataset for the ablation experiments used the goat face dataset. The experimental results are shown in Table 5. We used YOLOV7 (base) and backbone as a baseline. Row 2 (backbone + CIDM) is shown to outperform row 1 (backbone), where AP increases by 3.52%. In addition, the third row outperforms the first row (backbone), where AP increases by 5.73%, respectively. To investigate whether the combination of the CIDM and the FCM plays a role in this, we looked at row 4 (backbone + CIDM + FCM), which shows the highest performance of all settings, with an improvement of 8.07%.

For the statistical evaluation of the ablation experiments, we also used the same methodology described above, and Table 6 shows the sorting results of the different modules in comparison with the backbone. A chi-square value of $\chi^{2}$ = 12 and a *p*-value of *p* = 0.007 < 0.05 were obtained. This indicates that the individual modules present statistically significant differences between them. With the Nemenyi test, we calculated the critical distance, CD = 2.345, from which we can infer that our overall model CSTDNet is significantly different and performs better than backbone, verifying the validity of each module of CSTDNet. Table 6 shows the ranking of test results according to ablation test data.

**Figure 9:**
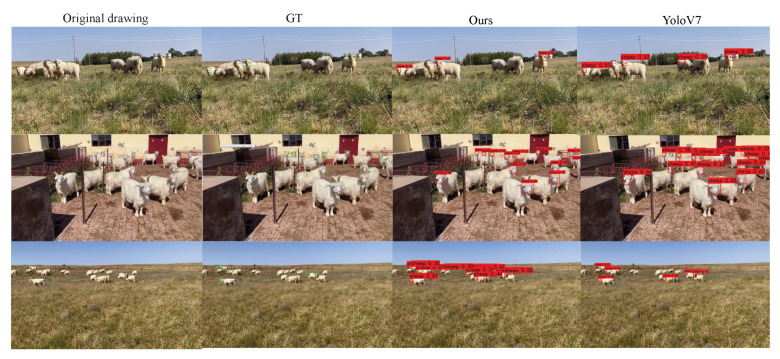
Predicted results.

Figure 9 shows the results of goat face recognition for both our method and YOLOV7. The first column is the image to be detected; the second column is Grouth True; the third and fourth columns are our and YOLOV7’s prediction results. It can be seen that our method accurately detects goat faces, and YOLOV7 suffers from some missed detections and a low detection frame IoU. This shows that, in comparison, our model can better detect small targets and is very accurate.

## 4. Discussion

This study aimed to use deep learning techniques to solve the problem of difficult localization and detection of goat faces in real livestock farm environments. First, we constructed a dataset of Alba velvet goat facial images and then developed a small target goat face detection network model. However, there are still some challenges and limitations to be addressed.

First, compared with other animals, goats have fewer facial texture features, which makes their face detection more susceptible to interference, such as complex sampling environments, lighting conditions, and different goat poses. This poses a challenge for accurately detecting goat faces in realistic scenarios. In addition, the lack of publicly available standardized datasets for goat face detection limits the evaluation and comparison of different detection methods. Future research should focus on constructing comprehensive and representative datasets specifically for goat face detection.

Furthermore, although our study demonstrated good results for goat facial detection, it is critical to apply our method to other goat species and validate its performance. Different goat species may exhibit differences in facial characteristics, and further investigation and adaptation of the detection model is needed to ensure its validity across different goat populations.

Practical applications of goat facial detection also need to consider challenges such as occlusion and different environmental conditions. Accurate detection of goat faces under partially obscured or challenging lighting conditions remains an important area for future improvement.

While this research helps advance goat face detection using deep learning, it is critical to address the above limitations and challenges. Further research should focus on developing robust detection models that can handle variations in facial features, incorporate different datasets, and address challenges encountered in real-world livestock management scenarios. These advances will help develop intelligent management systems that enhance the identification, tracking, and welfare of goat in modern livestock farms.

## 5. Conclusions

The experimental results show that the detection accuracy is low on small-target goat faces when directly using traditional target detection algorithms. In this paper, we proposed a goat face detection model that combines contextual information and feature-fusion complementary modules to solve the above problem. By using the target’s contextual information to provide more background and semantic information, and fusing the feature maps generated at different stages, the model performance is significantly improved and shows good robustness, reducing the impact of small targets in goat face target detection and providing a basis for the subsequent development of the intelligent management of goat in modern pastures.

## Figures and Tables

**Table 1:** Comparison of experimental results.

Module	P (%)	R (%)	F1	AP (%)
CenterNet [31]	88.02	52.36	0.66	71.51
EfficientDet [32]	95.55	22.0	0.36	39.77
SSD [33]	89.45	47.42	0.57	57.28
FASSD [11]	**98.41**	21.9	0.35	36.36
FCOS [34]	84.80	63.56	0.73	74.69
RetinaNet [35]	94.37	17.55	0.30	31.40
YOLOV5 [21]	84.47	51.22	0.63	63.45
YOLOV7 [20]	85.71	69.13	0.77	77.9
Ours	90.42	**75.93**	**0.83**	**85.97**

Bold is the maximum value in a single column.

**Table 2:** Ranking of small target detection results based on comparative test data.

Module	P	R	F1	AP	Average
CenterNet [31]	3	4	4	4	3.75
SSD [33]	2	6	6	6	5
FCOS [34]	5	3	3	3	3.5
YOLOV5 [21]	6	5	5	5	5.25
YOLOV7 [20]	4	2	2	2	2.5
Ours	1	1	1	1	1

**Table 3:** Normal target comparison experimental results.

Module	P (%)	R (%)	F1	AP (%)
CenterNet [31]	88.62	53.70	0.67	72.83
EfficientDet [32]	**95.60**	24.57	0.39	42.13
SSD [33]	88.02	49.48	0.63	71.73
FASSD [11]	92.41	31.17	0.46	48.52
FCOS [34]	93.02	**90.03**	0.92	94.04
RetinaNet [35]	94.69	19.85	0.33	33.79
YOLOV5 [21]	90.76	76.73	0.83	86.51
YOLOV7 [20]	95.13	89.47	**0.94**	**95.72**
Ours	94.52	88.75	0.92	94.59

Bold is the maximum value in a single column.

**Table 4:** Ranking of normal-sized target detection results based on comparative test data.

Module	P	R	F1	AP	Average
CenterNet [31]	6	5	5	5	5.25
SSD [33]	5	6	6	6	5.75
FCOS [34]	3	1	2.5	3	2.375
YOLOV5 [21]	4	4	4	4	4
YOLOV7 [20]	1	2	1	1	1.25
Ours	2	3	2.5	2	2.375

**Table 5:** Results of ablation experiments.

Module	P(%)	R(%)	F1	AP(%)
backbone	85.71	69.13	0.77	77.9
backbone + CIDM	88.78	73.17	0.8	81.42
backbone + FCM	88.84	74.22	0.82	83.63
backbone + CIDM + FCM	**90.42**	**75.93**	**0.83**	**85.97**

Bold is the maximum value in a single column.

**Table 6:** Ranking of test results according to ablation test data.

Module	P	R	F1	AP	Average
backbone	4	4	4	4	4
backbone + CIDM	3	3	3	3	3
backbone + FCM	2	2	2	2	2
backbone + CIDM + FCM	1	1	1	1	1

## Data Availability

The data presented in this study are available on request from the corresponding author.
